# A Macroporous TiO_2_ Oxygen Sensor Fabricated Using Anodic Aluminium Oxide as an Etching Mask

**DOI:** 10.3390/s100100670

**Published:** 2010-01-19

**Authors:** Chih-Cheng Lu, Yong-Sheng Huang, Jun-Wei Huang, Chien-Kuo Chang, Sheng-Po Wu

**Affiliations:** 1 Institute of Mechtronic Engineering, National Taipei University of Technology, Taipei 106, Taiwan; E-Mails: t7408047@ntut.edu.tw (Y.-S.H.); cooljunwei@hotmail.com (J.-W.H.); t7408021@ntut.edu.tw (S.-P.W.); 2 Graduate Institute of Mechanical and Electrical Engineering, National Taipei University of Technology, Taipei 106, Taiwan; E-Mail: chiean-kuo@yahoo.com.tw

**Keywords:** anodic aluminium oxide (AAO), macroporous, MEMS, TiO_2_ gas sensor

## Abstract

An innovative fabrication method to produce a macroporous Si surface by employing an anodic aluminium oxide (AAO) nanopore array layer as an etching template is presented. Combining AAO with a reactive ion etching (RIE) processes, a homogeneous and macroporous silicon surface can be effectively configured by modulating AAO process parameters and alumina film thickness, thus hopefully replacing conventional photolithography and electrochemical etch methods. The hybrid process integration is considered fully CMOS compatible thanks to the low-temperature AAO and CMOS processes. The gas-sensing characteristics of 50 nm TiO_2_ nanofilms deposited on the macroporous surface are compared with those of conventional plain (or non-porous) nanofilms to verify reduced response noise and improved sensitivity as a result of their macroporosity. Our experimental results reveal that macroporous geometry of the TiO_2_ chemoresistive gas sensor demonstrates 2-fold higher (∼33%) improved sensitivity than a non-porous sensor at different levels of oxygen exposure. In addition, the macroporous device exhibits excellent discrimination capability and significantly lessened response noise at 500 °C. Experimental results indicate that the hybrid process of such miniature and macroporous devices are compatible as well as applicable to integrated next generation bio-chemical sensors.

## 1. Introduction

With the rapid evolution of microelectronic and microelectromechanical systems (MEMS) technologies, recent industrial products show a clear trend towards miniaturization and neatness. In addition to the traditional CMOS process, MEMS has become a key technique for shrinking device and system dimensions, and the most emerging and cutting-edge field in sensor research during the past two decades. It has effectively reduced device dimensions, lowered material volume and element cost, and more importantly, lessened operational power consumption if necessary. Besides, it also demonstrates the capability of batch processing to produce numerous microsensors and microsystems, which ensures the functionality and availability of CMOS- or silicon-based devices. Relevant studies on the use of SOI technology platform as a full CMOS-MEMS approach for low-power gas detection [1–3] were carried out. These are quite advantageous in the gas sensors development track. However, one still can see that these micro-scale gas sensors may face poorer sensitivity or response characteristics compared to bulk ones, due to significant reduction of the surface reaction area in chemically sensitive materials. Hence the use of porous or macroporous materials to enhance the deficient sensitivity and response speed of MEMS gas sensors is very popular because the sensor sensitivity is proven to be proportional to the specific surface area [4].

Of all gas sensors, chemoresistive gas sensors deposited with metal oxide thin films have been well developed and are widely employed in industrial applications and environmental control. A number of metal oxide semiconducting materials such as ZnO, SnO_2_, TiO_2_, In_2_O_3_ and WO_3_ have been studied to detect hazardous and combustible gases [5–10]. Metal oxide bulk films were mainly deposited by physically dispersion or physical vapour deposition (PVD) on the active areas of miniature sensors. However, considerable power losses due to high-temperature operation and poor sensitivity resulting from reduced active area of the gas-sensitive films are still problematic issues. Recently, along with widespread applications of nanostructural materials such as nanotubes, nanoparticles, nanobelts and nanowires, their significant nanoporosity and extensive surface areas could provide promising solutions to improve sensor sensitivity and response characteristics. Alternatively, one may use mesoporous particles as the gas-sensitive film with dielectrophoresis (DEP) processes [11]. However there was room for improvement of the immobilisation method and adhesion strength between the active material and device. These nanostructural particles may separate from the sensor surface because of external force or environmental factors and this can deteriorate a sensor’s lifetime. On the other hand, conventional poration approaches to fabricate a porous surface mainly include lithographic patterning [12,13] and electrochemical hydrofluoric acid etch [14]. However, the former is limited by lithographic resolution and expensive cost; in the latter it is still difficult to regulate uniform pore size and density, though it was able to produce nanoporous materials.

To date, there have been a number of continuous investigations on the anodic aluminium oxide (AAO) process since Keller *et al.* [15] discovered porous aluminium oxide layers in 1953 by immersing aluminium metal in electrochemical acid solutions. The AAO layer, essentially a cluster of alumina nanopores, features macroporous (*i.e.*, pore diameter >50 nm), self-organized, highly ordered and homogeneous pore arrays [16–21] and can be prepared directly on aluminium metal sheets, or on silicon substrates by coating high-quality aluminium metal films [22–25]. It has been known that the adoption of acid electrolytes including C_2_H_2_O_4_ [17,18], H_3_PO_4_ [19,20], H_2_SO_4_ [23] is able to produce AAO nanopores with more satisfactory poration rates and pore uniformity than alkali electrolytes. AAO nanopores normally range from tens of nanometers to hundreds of nanometers in diameter, depending on the electric or chemical parameters modulated and thus have larger specific surface areas than plain thin films with the same dimensions. It is considered a simple, low-temperature, cost-effective and MEMS-compatible process, which makes it possible to act as a platform for thin film-based sensors, solar cells, fuel cells or quantum dot applications.

Herein we present a novel approach to produce macroporous metal oxide gas sensors which combines the AAO thin film and microfabrication processes. As can be seen, the AAO layer features highly ordered and homogeneous alumina nanopores that make it suitable as a dry-etching mask on the silicon substrate to generate a macroporous surface. The geometric features such as pore diameter, pore density and pore depth can be experimentally controlled by modulating AAO process parameters such as aluminium film quality, applied potential and electrolyte conditions. Experimental work including preparation of AAO process and fabrication flow of this gas sensor is introduced first, followed by a description of the characteristics of the macroporous surface and as-deposited TiO_2_ thin films. Response characteristics and sensitivity of macroporous and non-porous TiO_2_ films to different oxygen concentrations at room temperature (31 °C) and a high temperature (500 °C) have been provided and compared. All these experimental results verify the promising feasibility of this class of macroporous TiO_2_ thin film gas sensors fabricated using such well-developed techniques.

## 2. Experimental

### 2.1. Preparation of the AAO Process

**Figure 1.**
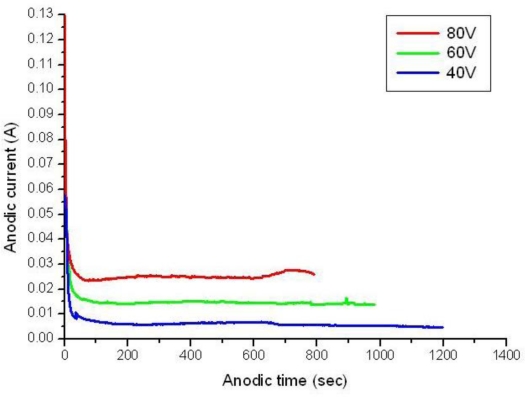
Anodic current monitoring during the AAO process under various voltage.

**Figure 2.**
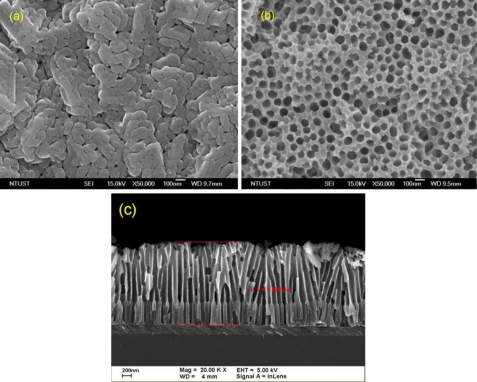
SEM images of the AAO layer: (a) processed under 60 V, 10 min, (b) processed under 60 V, 40 min (the pore-widening step), (c) illustrated with cross-section before the pore-widening step.

Before the beginning of the AAO process, the silicon wafer was deposited with an aluminium thin film of 1 μm RF sputtered onto the front side of the silicon substrate. The AAO process was then carried out by using 0.3 M oxalic acid solution at 4 °C in an electrochemical cooling tank for 90 min. The electrochemical reaction was conducted under dc 40–60 volts by a Keithley 2000 sourcemeter and the corresponding anodic current during the AAO process was closely monitored by a computer connected to a LabVIEW^®^ data acquisition adaptor. The anodic I-V curve exhibited a significant drop to very low current within 100 s during when the aluminium oxidation procedure started, and ended at various time points under different voltage, as shown in Figure 1. After the oxidation process, the alumina thin film was immersed in a 5% phosphoric acid solution for 4 h at room temperature. This step was relatively vital to produce a homogeneous pore diameter distribution and pore-widening effect in the alumina nanopore array. In addition, the alumina layer and the barrier layer located at the base of the alumina layer were further thinned using a 30% phosphoric acid solution at room temperature to make it suitable to act as an etch mask for later use. Compared with the AAO methods developed earlier, the AAO process developed in our laboratory was adjusted and simplified so that merely one pore-widening step was required and the barrier layer in the bottom part could be easily removed. The as-fabricated AAO films in Figure 2 exhibit anodic oxidation results before and after the alumina pore-widening step.

### 2.2. Manufacture of Macroporous Silicon

**Figure 3.**
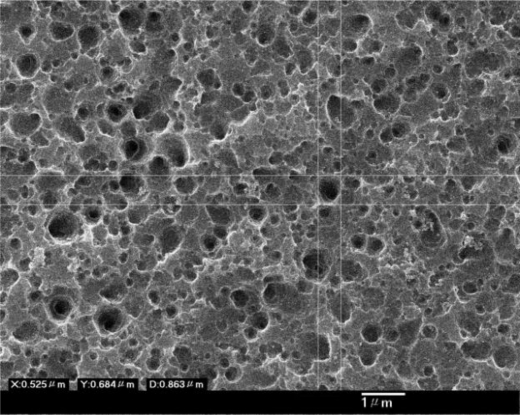
SEM image of the macroporous Si surface after RIE process and removal of the alumina layer.

With thinned AAO films, it is very feasible to create porous silicon, silicon oxide or any other treatable materials. The thinned layer of anodic aluminium oxide readily acts as an dry-etch mask for an isotropic reactive ion etch (RIE) process, enabling the silicon surface under the alumina mask to generate macroporous pores estimated 100–700 nm in diameter. The alumina nanopore array works as a competent etch mask due to the good selectivity relative to silicon in an RIE process with CF_4_:Ar:O_2_ = 5:0.5:0.5 in sccm and a pressure of 90 mTorr. The thin barrier layer at the base of the alumina mask can be removed first. On completion of RIE process, the alumina mask was removed by a 30% H_3_PO_4_ solution at 60 °C for 1 h. A scanning electron microscopic (SEM) micrograph of the macroporous silicon surface after an RIE process is shown in Figure 3.

### 2.3. Design and Fabrication of Macroporous TiO_2_ Gas Sensors

**Figure 4.**
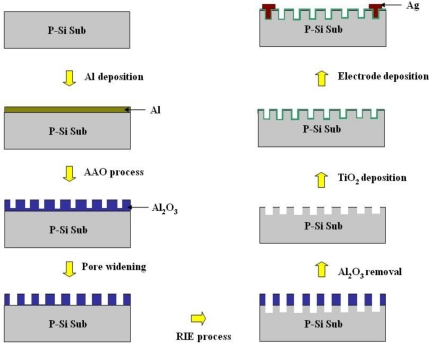
The processing schematic of a macroporous TiO_2_ gas sensor.

To fabricate macroporous TiO_2_ gas sensors, the integration of AAO and RIE etch processes was performed. The sensor was manufactured by our revised AAO process mentioned before and microfabrication techniques. The process flow for the macroporous TiO_2_ sensor is shown in Figure 4. A similar concept of sensor fabrication was also reported by Lu and Chen [26] in 2009; however, the AAO layer was not removed and the gas-sensitive TiO_2_ film was directly grown onto it. During the AAO electrochemical process, according to our experimental practice, the alumina layer was inclined to peel off or get damaged by other oxidation steps, which may not afford sufficient adhesion to the substrate. It is our belief that the AAO layer as the dry-etch mask can produce a porous silicon surface, and after its removal, a reliably adhesive TiO_2_ macroporous thin film deposited onto the silicon surface can be highly guaranteed. The RIE process readily etched the silicon substrate surface to form nano-scale pores ranging from 100 nm to 750 nm in diameter. After an ultrasonic cleaning step, the gas-sensitive layer of polycrystalline TiO_2_ was deposited onto the porous silicon surface to configure a macroporous TiO_2_ thin film by a RF sputtering process. In consideration of the nanopore diameter distribution on the silicon surface, the TiO_2_ thin film with a thickness of 50 nm was selected to verify its sensitivity and response properties. Finally, silver electrodes were deposited on the macroporous TiO_2_ thin film by a lift-off technique. The material characterization and geometric profile of the porous TiO_2_ thin film will be shown and discussed later.

### 2.4. Gas Characterization of the Macroporous TiO_2_ Sensor

**Figure 5.**
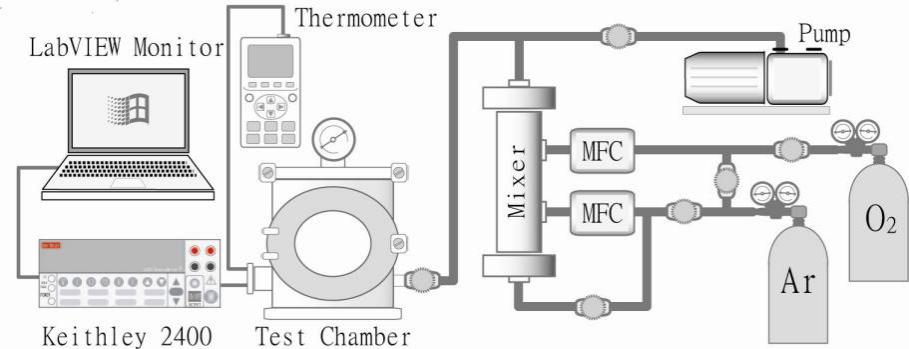
A configuration schematic of the gas test system.

A gas test system was utilised to characterise various responses to toxic or hazardous gases. This system basically includes two-channel gas pipelines, a gas mixer and a test chamber with high-precision mass flow controllers (MFCs). Other facilities such as a mechanical vacuum pump and a vacuum gauge, a multi-channel thermometer, a Keithley 2400 sourcemeter and a LabVIEW^®^ data acquisition card were also appropriately linked to this system. A configuration diagram of the gas test system is shown in Figure 5.

Initially, argon carrier gas was introduced into the system to evacuate or dilute the air inside the system as oxygen in air may affect the accuracy of the response to an analyte gas. The fabricated TiO_2_ sensor was placed in the test chamber and characterised under exposure to a mixture of analyte and carrier gases in atmosphere pressure (gas adsorption phase), then evacuated to a low pressure of 10^−3^ Torr or heated up by a heater (gas desorption phase), and simultaneously measured by *in situ* resistance readout from the electrodes using a LabVIEW^®^ software interface.

## 3. Results and Discussion

The surface geometry of the macroporous TiO_2_ sensor was verified by a SEM image, and its X-ray diffraction (XRD) pattern was analyzed to examine the thin film crystalline structure. Furthermore, discussions on AAO process experiments and parametric operation were reported. Atomic force microscopy (AFM) was also employed to investigate the aspect ratio of pore depth to pore diameter in the fabricated nanopores that were not clearly shown in the SEM images. Next, to explore the gas-sensitive effect enhanced by the macroporous TiO_2_ surface, plain and AAO-based porous thin films of 50 nm thick were deposited and made into gas sensors. Oxygen analyte gas mixtures of different concentrations mixed with an argon carrier gas was prepared and supplied to measure chemoresistive response and sensitivity due to analyte gas exposures at the room temperature of 31 °C and an elevated temperature of 500 °C, respectively. All gas tests were conducted at ambient atmosphere pressure, room temperature of 31 °C and 65% RH. As usual, the sensor sensitivity to an analyte gas can be defined as *S* = Δ*R*/*R*_0_ × 100%, where *R*_0_ is the baseline resistance in the presence of the carrier gas without an analyte gas and Δ*R* is the difference between *R*_0_ and the resultant resistance after the analyte gas appears in the test chamber and an equilibrium is reached. All test results were compared, analyzed and summarized.

### 3.1. Characterization of the Porous TiO_2_ Thin Film

**Figure 6.**
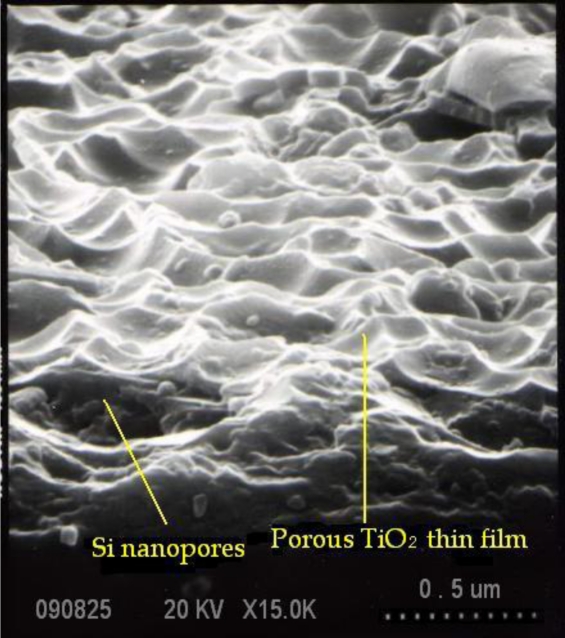
SEM image of the macroporous TiO_2_ thin film deposited on the silicon substrate.

Figure 6 exhibits an SEM diagram of the macroporous geometry of the 50-nm TiO_2_ thin film after AAO process and sputtering deposition.

**Figure 7.**
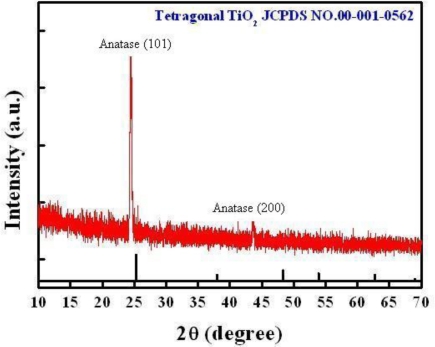
XRD pattern analysis of the porous TiO_2_ thin film.

As one can see, the TiO_2_ layer successfully covers the porous silicon substrate and duplicates the wavy geometry underneath. From this evidence, it is shown that the specific surface area of the TiO_2_ thin film is significantly increased compared to a conventionally deposited plain (or non-porous) thin film. Next, X-ray diffraction (XRD) pattern analysis was carried out to characterize the composition of as-deposited TiO_2_ thin film. The XRD pattern was obtained using a Bede/D1 diffractometer with Cu Kα radiation (1.541 Å) made by MAC science Ltd. Usually, it is noted that small-angle XRD analysis is typically employed for thin films while large-angle XRD analysis is for bulk materials. As one can see a full-range angle XRD scan was employed that confirmed prominent intensity peaks at 2-theta degree of 24.97° and 43.85°, as shown in Figure 7, which can be explained as the crystalline structure of anatase instead of rutile after film deposition based on JCPDS database and considered suitable for gas detection [27]. No annealing process was applied to the TiO_2_ layer deposited in our experiments.

### 3.2. Modulation and Analysis of AAO Porosity

**Figure 8.**
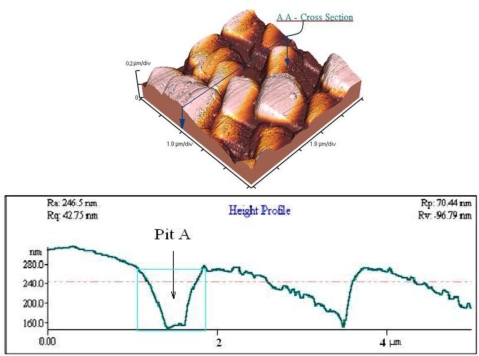
The porous TiO_2_ thin film of AFM measurement.

In the anodic aluminium oxidation process, the deposition of quality aluminium films can mostly dominate the orderly geometry of the alumina nanopore array. Some operating steps such as wafer cleaning, particle removal, deposition parameter setup and substrate materials are closely associated with the successful implementation of AAO layers. A common failure encountered is AAO film peel-off in the beginning or half way through the process. Our experimental results indicate that higher anodic voltages, for instance, greater than 100 V tend to cause peel-off or deionization of the aluminium film in oxalic acid solution. Therefore, the hard anode mode with anodic voltage ranging from 40 V to 80 V is commonly employed. The higher the working voltage, the shorter the AAO reaction period, as shown in Figure 1. Such an operation may result in a larger aspect ratio of depth to width (>16) in the alumina nanopore array, which is not advantageous for use as a mask for an RIE etching process. However, the thickness of the alumina layer can be further reduced by a 30% H_3_PO_4_ solution at room temperature and this makes a suitable RIE etch mask with a lower aspect ratio (<5). Also, appropriate manipulation of anodic voltage and current has a vital impact on determination of nanopore diameters. It is noted that nanopores with larger diameters can be induced at higher anodic voltages and *vice versa*. Moreover, the Joule heat generated in the anodic oxidation is able to raise the solution temperature and thus cause unstable electrochemical AAO reactions, leading to peel-off of the AAO film. A magnetic stirring rotator is placed in the solution to diminish the undesired thermal effect. To characterize the as-etched silicon surface, an atomic force microscope was employed to describe the surface geometry. Figure 8 illustrates the AFM geometry showing the 2-D porous silicon surface and a cross-sectional profile along Y-direction (not to scale). The profile indicates that a nanopore etched on the silicon surface has an estimated aspect ratio of 0.35 at pit A in Figure 8, which is suitable for thin film deposition and coverage.

### 3.3. Oxygen Response at Room Temperature (31°C)

**Figure 9.**
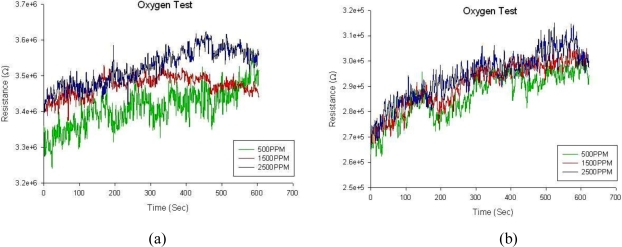
Temporal resistance response *vs.* different oxygen concentrations at room temperature of 31 °C with (a) a plain (non-porous) TiO_2_ film, and (b) a macroporous TiO_2_ film. The film thickness is 50 nm.

It’s been recognized that the resistance change of metal oxide thin films such as TiO_2_ or SnO_2_ behaves as an *n-type* semiconductor in an oxygen-contained environment [28]. At high temperatures the temporal resistance change under gas exposure is caused by analyte gas molecules that react irreversibly with electrons or holes of the TiO_2_ lattice vacancies. A surface depletion region referred to as the Debyé length is thus formed on TiO_2_ surface and affects the Schottky barrier, which is dominated by charge carrier concentrations. The Schottky barrier is exponentially associated with material conductance and the conductance variation is proportional to gas concentrations as well. In oxygen or other oxidizing gases, it was shown that the resistance in TiO_2_ can be increased by exposing to rising concentrations of oxygen, and vice versa in reducing gases. The macroporous TiO_2_ thin film, as a result, represents a higher specific surface area and contributes higher charge carrier concentrations that can enhance the conductance, and thus demonstrates decreased resistance when compared with a plain TiO_2_ thin film. A number of previous studies disclosed that most TiO_2_-based sensors must operate over 400 °C for better stability and performance [4–6]. However, to examine the impact of macroprorosity to TiO_2_ sensors, a series of gas tests addressing gas response to different oxygen concentrations for porous and non-porous TiO_2_ sensors were carried out. These TiO_2_ gas sensors were tested at room temperature of 31 °C, 65% RH and 1 atm in a completely sealed chamber with a gas inlet from the MFCs and the mixer, and a gas outlet to ambient air. With precise MFCs one can modulate various gas flow rates to produce expected concentrations of the analyte gas. Figure 9 shows the resistance change of the sensor in response to 500 ppm, 1,500 ppm and 2,500 ppm of oxygen gas. It is observed that resistance signals were highly unstable and fluctuated, despite the increase of oxygen concentrations. Higher oxygen concentrations up to 1% even did not help diminish the phenomenon. It was also shown that three temporal response curves of different oxygen concentrations shown in Figure 9 were very close to each other and discrimination ability was limited at room temperature regardless of TiO_2_ macroporous films. However, the ascending trend of resistance variation with elapsed time was clearly verified and still consistent with oxidized characteristics of the TiO_2_ thin film. These indistinct resistance responses and significant noise are not unexpected because experiments were operated at room temperature and could be attributed to wide-gapped Ag electrodes in part.

### 3.4. Oxygen Response at High Temperature (500 °C)

**Figure 10.**
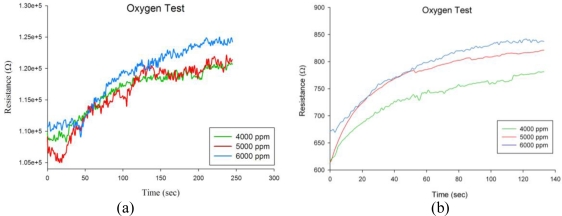
Temporal resistance response *vs.* different oxygen concentrations at a high temperature of 500 °C with (a) a plain (non-porous) TiO_2_ film, and (b) a macroporous TiO_2_ film. The film thickness is 50 nm.

Applying most test conditions of the room-temperature experiment, porous and non-porous sensors were characterized instead at a high temperature of 500 °C. The experiments were carried out to reveal resistance change of the sensors *vs.* 4,000 ppm, 5,000 ppm and 6,000 ppm of oxygen gas, as illustrated in Figure 10. It was shown that, unlike the unstable response in room-temperature tests, both temporal resistance changes to a constant oxygen concentration for both porous and non-porous devices became more stable and less noisy, particularly prominent for the porous type sensor. A significant high temperature effect on improving the resistance response of TiO_2_ thin films has been demonstrated when compared to the room-temperature operation. Our experimental results also showed that the TiO_2_ resistance at 500 °C is lower than that at 31 °C. Two things may explain this phenomenon. One is the *n-type* semiconducting model at high temperatures; *i.e.*, the higher the temperature, the lower the resistance. The second is that TiO_2_ resistance can be also affected by humidity factor and it was revealed that the resistance of TiO_2_ thin film lessens while relative humidity falls [29], particularly when operating at high temperatures. In addition, the porous TiO_2_ sensor obviously proved better discrimination ability for oxygen with different concentrations than the non-porous one.

In summary, the response behaviors suggested that TiO_2_ gas-sensing films should be considered an *n-type* semiconductor when exposed to an oxidizing gas. More interestingly, we converted the resistance response characteristics into charts expressing sensitivity *versus* oxygen concentration.

**Figure 11.**
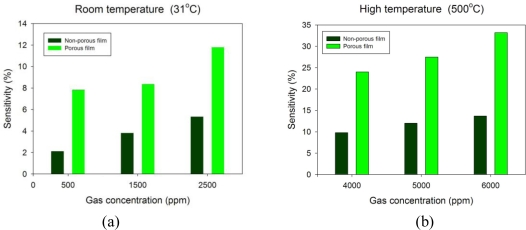
Comparative charts of sensitivity *vs.* oxygen concentrations with respect to non-porous and porous TiO_2_ thin film sensors at (a) room temperature of 31 °C and (b) a high temperature of 500 °C.

In Figure 11 it is clearly observed that the macroporous TiO_2_ sensor can effectively elevate sensor sensitivity by more than 2-fold (∼33%) when compared to the non-porous TiO_2_ sensor at different levels of oxygen exposure, both at room temperature and high temperatures. Also, one can clearly see that both resistance response and sensitivity of the sensor are increasingly proportional to rising oxygen concentrations, delivering an informative and favourable behaviour for analyte calibration.

## 4. Conclusions

A new approach to fabricate macroporous TiO_2_ oxygen sensors utilizing anodic aluminium oxide nanostructures as an effective dry-etch mask for production of a porous silicon substrate is described. This new method is mainly developed for generating a higher specific surface area that is believed to reduce sensor dimensions and enhance sensor sensitivity by integrating low-temperature AAO and microfabrication process. With positive experimental results, it is noteworthy that the AAO-fabricated TiO_2_ porous gas sensor can not only lessen response noise, but also significantly enhance sensor sensitivity by a significant 2-fold increase (∼33%) compared to that of a non-porous TiO_2_ sensor at 500 °C for oxygen detection. The macroporous TiO_2_ sensors are able to achieve successful oxygen discrimination in terms of distinct resistance response and low measurement noise at high temperatures. We could attribute these advantages to an increased specific surface area in macroporous TiO_2_ thin films fabricated using the proposed hybrid process.
